# Insights into the relationship between eye movements and personality traits in restricted visual fields

**DOI:** 10.1038/s41598-024-60992-w

**Published:** 2024-05-04

**Authors:** Kuangzhe Xu

**Affiliations:** Institute for Promotion of Higher Education, Hirosaki University, Aomori, 036-8560 Japan

**Keywords:** Psychology, Human behaviour

## Abstract

Previous studies have suggested behavioral patterns, such as visual attention and eye movements, relate to individual personality traits. However, these studies mainly focused on free visual tasks, and the impact of visual field restriction remains inadequately understood. The primary objective of this study is to elucidate the patterns of conscious eye movements induced by visual field restriction and to examine how these patterns relate to individual personality traits. Building on previous research, we aim to gain new insights through two behavioral experiments, unraveling the intricate relationship between visual behaviors and individual personality traits. As a result, both Experiment 1 and Experiment 2 revealed differences in eye movements during free observation and visual field restriction. Particularly, simulation results based on the analyzed data showed clear distinctions in eye movements between free observation and visual field restriction conditions. This suggests that eye movements during free observation involve a mixture of conscious and unconscious eye movements. Furthermore, we observed significant correlations between conscious eye movements and personality traits, with more pronounced effects in the visual field restriction condition used in Experiment 2 compared to Experiment 1. These analytical findings provide a novel perspective on human cognitive processes through visual perception.

## Introduction

In contemporary society, elucidating and understanding the factors that influence human behavior and decision-making is a crucial challenge. Among these factors, visual information processing plays a central role when interacting with the environment and engaging with others. Specifically, visual attention and eye movements play significant roles in individual cognitive processes, and the progress in comprehending how these factors impact an individual’s personality and decision-making continues.

Personality traits and eye movements have been extensively studied, particularly in the field of personality psychology. Research in this area aims to elucidate the impact of individuals’ inherent personality traits on their behavior and decision-making. Studies based on the Big Five model have defined fundamental personality traits, such as extraversion, agreeableness, conscientiousness, neuroticism, and openness^1–3^. It has been revealed that these traits play crucial roles in individuals’ daily lives. For instance, individuals with different personality traits exhibited variations in performance in different occupations^4^. Moreover, personality traits were found to influence executive functions in information retrieval, extending beyond the realm of work^5^. Furthermore, in social settings, individuals were observed to make personality judgments based on others’ facial traits or expressions^6,7^. In addition to facial features, a variety of social factors, such as age, gender, and the relationship with the observer, have been verified to attract the observer’s attention and draw their gaze^8^. Furthermore, it has been confirmed that a person’s personality traits are actually related to the formation of attention towards social situations, such as landscapes or objects^9^. Thus, personality traits not only serve as the starting point influencing people’s behavior but also act as the endpoint influencing impressions and evaluations of others. Understanding how individual differences in personality traits impact human behavior and decision-making provides essential insights into comprehending personality traits.

Research on eye movements is crucial for understanding the mechanisms of visual information processing and deepening our knowledge about behavior and cognitive processes. Previous studies have revealed associations between eye movement patterns and cognitive processes in various visual tasks^10,11^. In particular, eye movements when observing human faces have been shown to closely relate to the physiological response of brain waves (N170), indicating a strong connection^12,13^. Additionally, cultural influences have been suggested, as individuals from Eastern and Western cultures exhibit different eye movement patterns when observing faces, implying the impact of external factors, such as culture, on eye movements^14,15^. Numerous studies have demonstrated the sensitivity of eye movements to stimuli that are highly specific to humans, such as faces^16–20^ and bodies^21,22^. Studies related to saccadic trajectories, which are closely linked to eye movements, have revealed a diverse relationship with facial features in a social context^23^. It was shown that eye movements demonstrate a significant correlation when observing faces. Particularly, it was confirmed that eye movements are effective in the learning and processing of new faces^24^. Furthermore, leveraging insights gained from eye movement research has proven valuable in exploring therapeutic approaches for conditions like Autism Spectrum Disorder (ASD) and prosopagnosia (face blindness)^25–28^. These findings provide a foundational understanding of how eye movements influence human behavior and cognition, contributing to ongoing efforts to develop interventions for various disorders.

In recent years, a growing body of research has focused on the relationship between eye movements and personality traits. Starting from early studies investigating the potential reflection of individual personality traits in eye movements^29^, more recent research has suggested that eye movement data are also associated with the formation of individuals’ cognitive processes and emotional cognition^30–32^. For instance, in various impression-rating tasks that involve facial images, strong correlations were observed between personality traits and eye movements^33^. Additionally, tasks related to facial expression recognition revealed a robust correlation between personality traits and eye movements^34,35^. These research findings have advanced our understanding of how objective physiological indicators, such as eye movements, are related to subjective evaluation indicators like personality traits.

Nevertheless, an experiment involving impression-rating tasks, where observers received explicit observation instructions, reported that despite engaging in unfamiliar observation behaviors due to these instructions, certain relationships between eye movements and personality traits were almost identical to those observed in free observation scenarios^36,37^. This indicates that behavior was observed where eyes were unconsciously directed towards areas deemed “not to be looked at” or considered “information-free.” This result suggests the possibility that when humans observe their surroundings, conscious (when they are aware) and unconscious (when they are not aware) eye movements may blend together. Studies have also specifically focused on conscious eye movements. In one such study^38^, researchers explored the effects of conscious observation behaviors on judging facial expressions in videos depicting faces represented by landmarks. To simulate conscious observation behavior, an experimental setup was created where only a small area around the mouse cursor was visible. Participants observed the facial stimuli by moving the cursor and made judgments about the expressions. The results revealed significant correlations between personality traits and conscious observation behaviors. Understanding how specific visual observation behaviors under certain conditions relate to individuals’ conscious processes offers new insights into visual behavior research. However, in this study, the movement of the cursor was considered a conscious observation behavior, hence no eye movement recordings were taken. Furthermore, while the effects of conscious observation behaviors were examined, they were not compared with free observation scenarios, indicating a need for more in-depth exploration in this field. Although concerns exist about the impact of methods that restrict the observation range on face recognition^39^, reports suggest that in scenarios where the observation range can be freely moved, facial features are captured in a manner similar to free observation^38^. It is believed that an experimental design that minimally interferes with participants’ observational intent can significantly reduce the impact on face recognition.

In this study, we aim to delve deeper into understanding how conscious eye movements induced by visual field restrictions relate to an individual’s personality traits. Specifically, drawing inspiration from prior research^38^, we will observe faces under conditions of limited visual fields and conduct impression-rating tasks. We will analyze the relationships between personality traits and eye movements (a restricted visual field group and a free observation group) in different evaluation tasks using a hierarchical Bayesian model. By elucidating the relationships between eye movements under visual field restrictions and behavior and personality traits, we seek to enhance our understanding of visual behavior based on individual characteristics. We anticipate that this exploration will not only contribute to future clinical applications but also to cross-cultural studies and a more profound understanding of human behavior.

## Experiment 1

In Experiment 1, two conditions were established: one with a restricted observation and one without restrictions. We conducted a behavioral experiment for impression-rating tasks. The size of the restricted observation area was determined with reference to prior research^40^ and set as a circular region within 2 degrees above and below the center of gaze. All participants in this study were provided with a comprehensive explanation of the objectives, procedures, potential risks, and benefits of the research, and written informed consent was obtained. All methods in this research were carried out with approval from the Ethics Committee of Hirosaki University (Approval Number: 0002(2023)), in accordance with relevant guidelines and regulations.

### Equipment

The experiment utilized the Tobii Pro Fusion eye tracker (120Hz) from Tobii Inc. to record participants’ eye movements. The experiment design was created using Psychopy. The experiment used a MacBook Pro (OS: Big Sur) and a 24-inch LG monitor (1080p). To stabilize the participants’ head positions, we employed a chinrest (TKD-UK1) from NAMOTO Inc.

### Participant

The sample size for this study was set at a minimum of 30 participants, based on prior research^14,38,41^. We recruited the participants in the experiment from Hirosaki University, with a total of 42 university students (29 males). All participants had normal vision (including corrected vision), and the average age was 19.8 (SD = 2.32). The details and purpose of the experiment were explained to participants in advance, and all participants underwent the experiment after demonstrating a clear understanding of the procedures. As compensation for their participation, each participant received a gift card of 1000 yen.

### Stimuli

In this study, we used 50 facial images as stimuli, following the approach of previous studies^33,36,42^. All of these facial images feature frontal views with neutral expressions. The individuals who provided these images are university students aged 18-22. To minimize extraneous factors, the images were converted to black and white using Photoshop, ensuring uniform brightness and contrast. Additionally, non-facial components (such as clothing below the neck, excluding hair) were removed, and the final images were standardized to a size of 412 $\times$ 558. Consistent with the experimental methodology of prior research, we fixed 10 facial images, randomly selected in advance, for each impression-rating item.

### Procedure

All participants received an explanation and practice session before the start of the experiment. After gaining sufficient understanding of the content and procedures of the study, they proceeded to participate in the main experiment. The main experiment included two conditions: one with a restricted field of view and another without such restriction (details of the field restriction method are explained in the “Visual field restriction” Section). The procedures for the experiments under these two conditions were identical.

The experiment begins with the question presentation stage, in which an impression-rating question is presented. Participants confirm the question and, at their own timing, click the mouse to proceed to the stimulus observation stage. In the stimulus observation stage, for the restricted field-of-view condition, the cursor’s surroundings are masked, allowing visibility only in a specific area. Participants can move the cursor to observe the stimulus image. After 3 seconds of observation time, the session automatically transitions to the evaluation stage. In the unrestricted condition, there is no interference mask, and participants can directly observe the stimulus image for 3 seconds. In the evaluation stage, participants provide a 7-point rating for the impression question asked in the question presentation stage. The experiment comprises a total of 50 sessions, with stimulus images presented randomly.

The impression evaluation items were selected with reference to previous studies^1–4^, and we adopted the Big Five items (extraversion, conscientiousness, agreeableness, neuroticism, and openness) as the evaluation items. Participants first underwent a behavioral experiment under the condition of restricted visual fields, followed by a short break, and then participated in a behavioral experiment under the condition of unrestricted visual fields. Participants first took part in the experiment under conditions with restricted visual fields to prevent a potential issue. If the experiment with restricted vision were conducted after participants had initially memorized the features of stimulus images under free observation conditions, it might lead to observation behavior focused more on identifying memorized facial features than on evaluating impressions. To circumvent this problem, participants were first introduced to the experiment under conditions with restricted visual fields. After completing all experiments, participants responded to the TIPI-J questionnaire^43^ for a self-assessment of their personality traits.

### Visual field restriction

The distance between participants and the monitor was fixed at 60 cm using a chin rest. Regarding the visual field restriction, based on a previous study^40^, it was set within a range of 2 degrees above and below the central line of sight (the discrimination visual field). Considering the distance from the participant’s head (chin rest position) to the monitor, the radius of the visible area reflected on the monitor was approximately 80 pixels. The method for the experiment involving the visual field restriction followed a previous study^43^, in which circular areas with a 40-pixel radius were created around the cursor’s center. Furthermore, a 40-pixel Gaussian filter was applied to smooth the peripheral areas, ensuring a gentle edge.

### Data processing

Based on previous studies^36,44^, eye movement data recorded by the eye tracker were formatted into an analyzable data structure. Specifically, participants’ eye movement coordinate data (1920 $\times$ 1080) for a single stimulus image were adjusted to match the size of the stimulus image (412 $\times$ 558). The adjusted data was then subjected to a Gaussian filter (SD = 10) and converted into weighted data with a unified scale. Subsequently, using pre-created masks for facial features, eye movement data corresponding to each facial part was extracted (the face mask includes the positions of the eyes, nose, mouth, eyebrows, forehead, and glabella for each stimulus image). Finally, the data for each extracted facial part was divided by the area (in pixels), resulting in the calculation of the weight of eye movement per pixel for each facial part. Additionally, as this study focused on eye movements during the observation of stimulus images, the analysis employed data solely from the eye movement during the stimulus observation stage.

### Analysis

Based on the previous study^38^, we analyzed the relationship between personality traits and eye movements using the ZIB model constructed according to Bayesian rules. The detailed model is presented in Eqs. (1)–(6). Here, *G* represents eye movements, and *n* denotes the index of each region (comprising a total of 6 locations: the eyes, nose, mouth, eyebrows, glabella, and forehead). *P* represents the matrix set of five personality traits for each participant; *q* corresponds to the parameter estimating the coefficients of the Bernoulli distribution model, representing the probability of focusing on a specific region. The parameters *a*, *b*, and $\mu _k$ are associated with estimating coefficients of the Beta distribution model, directly related to assessing the degree of focus on a specific region (representing dwell time), and $r^{subj}$ and $r^{pic}$ indicate random effects for participants and stimulus images, respectively.

1 $$\begin{aligned}{} & {} G_{n}\sim ZIB (q_{n},a_{n},b_{n}) \end{aligned}$$

2 $$\begin{aligned}{} & {} ZIB (G_{n}|q_{n},a_{n},b_{n}) = {\left\{ \begin{array}{ll} Bern (0|q_{n}) &{} ( G_{n} = 0 )\\ Bern (1|q_{n}) \times Beta (G_{n}|a_{n},b_{n}) &{} ( G_{n} > 0 ) \end{array}\right. } \end{aligned}$$

3 $$\begin{aligned}{} & {} q_{n} =\frac{1}{1+exp (-(\alpha ^{bern}_{k}+\sum ^{5}_{k=1}\beta ^{bern}_{k}P_{k(n)}+r^{subj(Bern)}_{i}+ r^{pic(Bern)}_{j}))} \end{aligned}$$

4 $$\begin{aligned}{} & {} a_{n}=\phi \cdot \mu _{n} \end{aligned}$$

5 $$\begin{aligned}{} & {} b_{n}=\phi (1-\mu _{n}) \end{aligned}$$

6 $$\begin{aligned}{} & {} \mu _{n} =\frac{1}{1+exp (-(\alpha ^{Beta}_{k}+\sum ^{5}_{k=1}\beta ^{Beta}_{k}P_{k(n)}+r^{subj(Beta)}_{i}+r^{pic(Beta)}_{j}))} \end{aligned}$$

We employed the Rstan package to construct our Bayesian model and estimate its parameters^45–47^. The prior distribution for fixed effects adhered to a normal distribution with a mean of 0 and a standard deviation of 10. Meanwhile, the prior distribution for random effects followed a gamma distribution ($\alpha$ = 10, $\beta$ = 10). We ran each model with default Stan hyperparameter values: 4 chains, 1 thin, 2000 iteration steps, and 1000 warm-up steps. Consequently, we obtained 4000 MCMC samples.

To ensure the convergence of MCMC estimations, we calculated Rhat (${\hat{R}}$) for each parameter, a widely accepted criterion for convergence. In line with typical MCMC estimation practices, we deemed estimations as converged when the number of chains was three or more and the Rhat was below 1.1 for all parameters. Based on these criteria, we confirmed the convergence of all parameter estimations.

We utilized the highest density interval (HDI) to assess the statistical significance of the model estimation results^45,48^. The HDI, a variant of a confidence interval, identifies the region with the highest density in the Bayesian posterior distribution, typically using a 95% HDI. This methodology allows us to consider the uncertainty associated with the model parameters and determine the range of parameter values considered most credible within the posterior distribution. Employing this approach facilitated the evaluation of the significance of the estimation results, providing a comprehensive perspective for interpreting the model outcomes.

### Result

#### Free observation condition

Table 1 summarizes the significant results of eye movement and personality traits under the free observation condition. The Bernoulli model indicates whether specific facial features were observed, whereas the Beta model illustrates how much attention was given to particular facial features. When evaluating agreeableness, individuals with high conscientiousness tended not to look at the nose but exhibited a tendency to focus on the forehead. When evaluating conscientiousness, individuals with high neuroticism tended to look at the mouth but not at the eyebrows. Additionally, individuals with high conscientiousness did not focus on the eyes and nose. In evaluating extraversion, individuals with high conscientiousness tended to look at the eyebrows but not at the nose. When evaluating neuroticism, individuals with high conscientiousness tended to look at the eyebrows but not at the eyes and nose. Finally, when evaluating openness, individuals with high openness tended to focus on the eyes.

**Table 1:** Significant correlations between eye movements and personality traits (Free observation condition).

Model	Impression	Area	Predictor	Mean	95%HDI
Bernoulli	Agreeableness	Eye	Open.	1.479	0.414 to 2.627
		Nose	Consc.	− 0.805	− 1.560 to − 0.048
	Conscientiousness	Glabella	Extra.	− 1.251	− 2.602 to − 0.031
		Eye	Consc.	− 0.882	− 1.879 to − 0.029
			Open.	1.390	0.102 to 2.797
		Nose	Consc.	− 0.730	− 1.381 to − 0.159
		Mouth	Neuro.	0.451	0.015 to 0.841
	Extraversion	Eyebrow	Agree.	− 1.263	− 2.610 to − 0.131
			Consc.	1.244	0.020 to 2.774
		Nose	Consc.	− 0.592	− 1.176 to − 0.023
	Neuroticism	Eyebrow	Consc.	15.747	3.551 to 28.194
		Eye	Consc.	− 1.374	− 2.445 to − 0.332
		Nose	Consc.	− 0.634	− 1.253 to − 0.051
		Mouth	Neuro.	0.608	0.143 to 1.088
	Openness	Eye	Open.	5.001	0.881 to 10.288
Beta	Agreeableness	Forehead	Consc.	0.154	0.005 to 0.331
	Conscientiousness	Glabella	Neuro.	− 0.152	− 0.296 to − 0.010

#### Discrimination visual field condition

Table 2 summarizes the significant results of eye movements and personality traits in situations with restricted visual fields. The Bernoulli model and the Beta model respectively indicate whether specific facial areas were observed and to what extent. When evaluating agreeableness, individuals with high conscientiousness tended not to look at the nose but showed a tendency to look longer at the forehead. Additionally, individuals with high extraversion tended to look at the forehead, and those with high neuroticism tended to look at the forehead when evaluating openness. Moreover, individuals with high neuroticism tended to look at the nose when evaluating openness. Compared to the results of the free observation condition, the number of significant correlations notably decreased.

**Table 2:** Significant correlations between eye movements and personality traits (Discrimination visual field condition).

Model	Impression	Area	Predictor	Mean	95%HDI
Bernoulli	Agreeableness	Glabella	Extra.	1.710	0.259 to 3.403
		Forehead	Neuro.	2.043	0.022 to 4.320
		Nose	Consc.	− 1.447	− 3.198 to − 0.032
	Openness	Nose	Neuro.	0.803	0.004 to 1.666
Beta	Conscientiousness	Glabella	Consc.	0.123	0.003 to 0.234

#### Verify changes in eye movements through simulation

**Figure 1:**
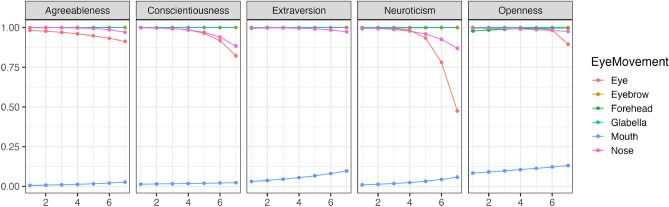
The impact of conscientiousness variations on eye movement during the free observation condition (Bernoulli distribution). The x-axis represents the conscientiousness scores, and the y-axis shows the probability of focusing on each part as estimated by the model. The closer to 1, the higher the probability of focus.

**Figure 2:**
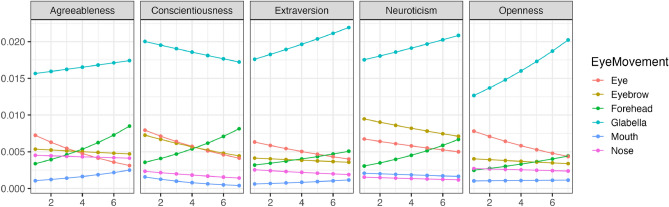
The impact of conscientiousness variations on eye movement during the free observation condition (Beta distribution). The x-axis represents conscientiousness scores, and the y-axis indicates the frequency of focus on each part as estimated by the model (following a beta distribution). The higher the value of Y, the higher the frequency of focus.

Using the estimated parameters from the model, we conducted simulations to verify changes in each eye movement for different impression evaluations. In this study, prioritizing the readability of results, confidence intervals and error bars were not included in the graphs, but the published result data does contain confidence intervals. Additionally, as an example explanation, we selected changes in conscientiousness with many correlations by referring to the results in Table 1. Henceforth, for consistency, all example explanations will focus on changes in conscientiousness. Further detailed results can be checked at the https://osf.io/8zfc5/. Figure 1 illustrates the trend of changes in whether each facial area was observed (Bernoulli distribution) during free observation with variations in conscientiousness. Figure 2 illustrates the trend of changes in how much each facial area was observed (Beta distribution) with variations in conscientiousness. Conscientiousness was scored on a 7-point scale from 1 to 7, whereas other personality traits were fixed with a score of 3. As a result, except for extraversion ratings, an increasing conscientiousness tended to be associated with a decreased tendency to look at the eyes. Moreover, all impression evaluations generally confirmed that individuals with higher conscientiousness tended not to look at the mouth. Conversely, excluding conscientiousness ratings, an increasing conscientiousness was associated with a tendency to look longer at the area between the eyebrows.

**Figure 3:**
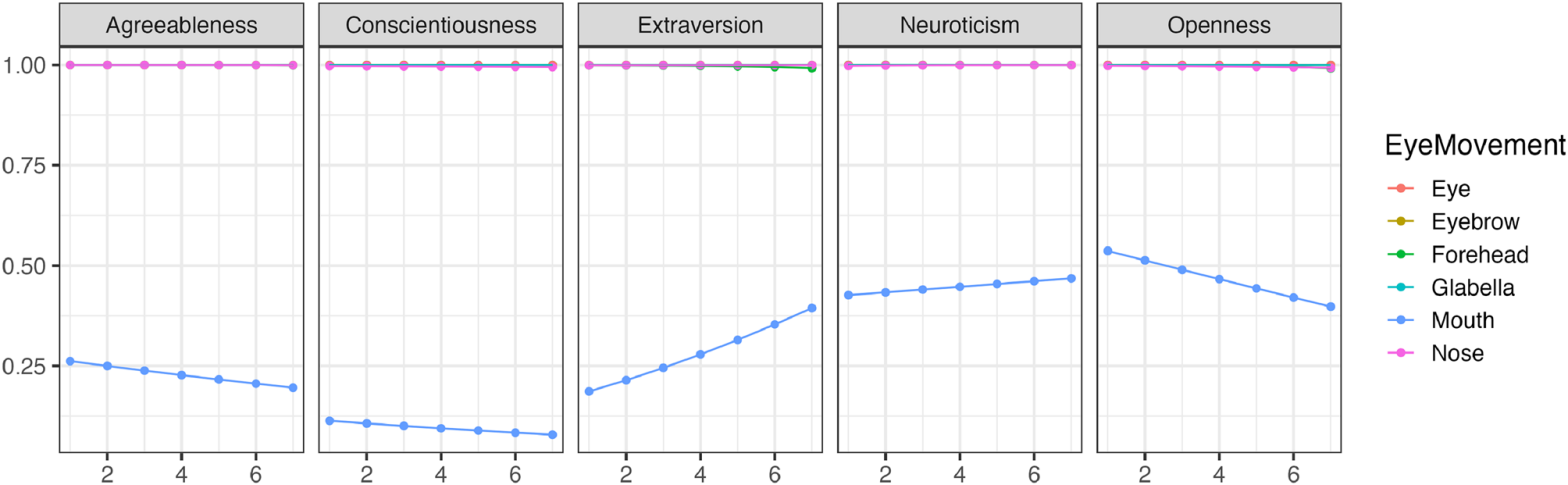
The impact of conscientiousness variations on eye movement during the discrimination visual field condition (Bernoulli distribution). The x-axis represents the conscientiousness scores, and the y-axis shows the probability of focusing on each part as estimated by the model. The closer to 1, the higher the probability of focus.

**Figure 4:**
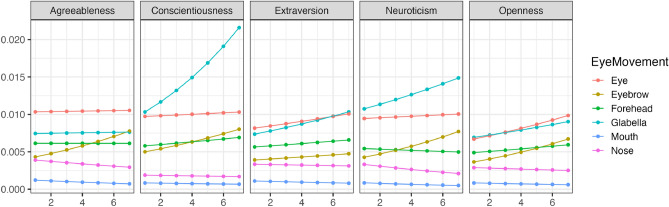
The impact of conscientiousness variations on eye movement during the discrimination visual field condition (Beta distribution). The x-axis represents conscientiousness scores, and the y-axis indicates the frequency of focus on each part as estimated by the model (following a beta distribution). The higher the value of Y, the higher the frequency of focus.

Figure 3 illustrates the changes in the perception of individual facial features (Bernoulli distribution) with variations in conscientiousness during restricted visual field conditions. Figure 4 demonstrates the trends in changes in how much each facial feature was observed (Beta distribution) with variations in conscientiousness. As a result, regardless of changes in conscientiousness, participants tended to look at all facial features, except for the mouth, with a high probability across all impression evaluations. Conversely, when evaluating conscientiousness, an increase in conscientiousness was associated with a tendency to look at the forehead region for most impression evaluations.

**Figure 5:**
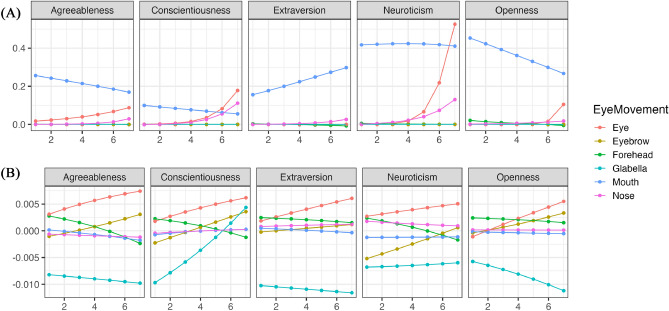
The relationship between the differences in estimated results between free observation and restricted visual field conditions and changes in conscientiousness. The x-axis represents the conscientiousness scores, the y-axis of A shows the difference in the probability of focusing on each part, and the y-axis of B shows the difference in the frequency of focusing on each part. If greater than 0, the restricted visual field condition is dominant; if less than 0, the free observation condition is dominant; the closer to 0, the less change there is.

To clarify the comparison between changes observed during free observation and restricted visual field conditions, the results from the simulations are displayed after subtraction (results of restricted condition–results of free condition). Figure 5’s panel (A) illustrates the difference between Figs. 1 and 3, while panel (B) displays the difference between Figs. 2 and 4. A value greater than 0 indicates dominance of the restricted visual field condition; a value less than 0 indicates dominance of the free observation condition; and the closer the value is to 0, the lesser the change observed. For additional results, please check the provided https://osf.io/8zfc5/. According to Fig. 5’s panel (A), individuals with higher levels of conscientiousness are significantly more likely to focus on the eyes in the restricted visual field condition when evaluating neuroticism. Conversely, in the context of assessing extraversion, these individuals are more inclined to focus on the mouth under the restricted visual field condition. As per Fig. 5’s panel (B), individuals with higher levels of conscientiousness are observed to spend significantly more time looking at the space between the eyebrows in the restricted visual field condition when assessing conscientiousness.

The estimation results of the impact of variations in other personality traits on eye movements can be found in the supplementary data (https://osf.io/8zfc5/). The results of how much was observed (Beta distribution) showed different patterns depending on the specific personality trait. For example, unlike the changing trend in conscientiousness during the visual field restriction, neuroticism and openness exhibited a tendency to not look at the forehead as they increased. However, the results of whether each region was observed or not (Bernoulli distribution) showed a consistent trend across all personality traits. The probability of looking at each region was very high in all cases.

### Discussion

The analysis results demonstrated a significant relationship between personality traits and eye movements during free observation, consistent with many aspects of prior studies. However, when the field of observation was restricted, there was a dramatic reduction in the number of significant correlations between personality traits and eye movements. In order to explore the cause, an examination of eye movement changes through model simulations was performed, which showed that attention was directed towards almost all facial regions. These findings suggest that the reduction in significant correlations could be attributed to the observation field being overly narrow and restrictive, potentially forcing participants to look at each facial area at least once. To more accurately capture conscious eye movements, the restricted field was modified, leading to the initiation of Experiment 2.

## Experiment 2

In Experiment 2, considering the findings from Experiment 1 and to tackle the problem of a too-narrow observation range, we adjusted the size of the restricted observation area and conducted Experiment 2 under conditions similar to Experiment 1. The observation area was set to extend 3 degrees above and below the gaze center. We chose not to expand the effective visual field beyond 3 degrees because we anticipated that further enlargement could reduce the effectiveness of the visual field restriction. The experimental setup and the methods for organizing and analyzing data remained unchanged. Similar to Experiment 1, all participants in this study were provided with a comprehensive explanation of the objectives, procedures, potential risks, and benefits of the research, and written informed consent was obtained. All methods in this research were carried out with approval from the Ethics Committee of Hirosaki University (Approval Number: 0002(2023)), in accordance with relevant guidelines and regulations.

### Participant

42 undergraduate students from Hirosaki University (25 females) participated in the experiment. All participants were in good health, with an average age of 20.1 (SD = 2.19). As compensation for their participation, each participant received a 1000-yen gift card.

### Result

#### Free observation condition

In Table 3, we summarize the significant results of eye movements and personality traits under the free observation condition. The Bernoulli model indicates whether specific areas were observed. The Beta model shows how much time was spent observing specific areas. When evaluating agreeableness, individuals with high neuroticism and openness tended not to look at the mouth. Individuals with high agreeableness and low conscientiousness tended to look at the nose. When assessing conscientiousness, individuals with high neuroticism tended not to look at the mouth. When evaluating extraversion, individuals with high conscientiousness tended not to look at the eyes, and individuals with high neuroticism tended not to look at the mouth. In the assessment of neuroticism, individuals with high neuroticism tended not to look at the mouth. When assessing openness, individuals with high neuroticism and openness tended to look at the forehead.

**Table 3:** Significant correlations between eye movements and personality traits (Free observation condition).

Model	Impression	Area	Predictor	Mean	95%HDI
Bernoulli	Agreeableness	Eyebrow	Extra.	9.425	1.343 to 19.704
		Nose	Agree.	8.357	1.113 to 16.799
			Consc.	− 11.827	− 22.150 to − 3.089
		Mouth	Neuro.	− 0.988	− 1.543 to − 0.489
			Open.	− 0.465	− 0.937 to − 0.017
	Conscientiousness	Mouth	Neuro.	− 0.840	− 1.426 to − 0.344
	Extraversion	Eye	Consc.	− 1.936	− 3.769 to − 0.314
		Mouth	Neuro.	− 0.740	− 1.192 to − 0.271
	Neuroticism	Mouth	Neuro.	− 0.808	− 1.362 to − 0.238
	Openness	Glabella	Neuro.	5.552	0.539 to 11.340
			Open.	4.865	0.393 to 9.323
		Mouth	Neuro.	− 0.699	− 1.154 to − 0.207
Beta	Neuroticism	Mouth	Neuro.	− 0.424	− 0.782 to − 0.092
			Open.	− 0.247	− 0.505 to − 0.003

#### Effective visual field condition

In Table 4, we compile the significant results of eye movements and personality traits under the condition of a restricted visual field. The Bernoulli model and Beta model respectively indicate whether specific areas were observed and how much time was spent observing them. When evaluating agreeableness, individuals with high neuroticism tended not to look at the forehead and eyes but showed a tendency to look longer at the forehead. Additionally, individuals with high openness tended not to look at the forehead and, simultaneously, tended not to look for an extended period at the mouth. When assessing conscientiousness and extraversion, individuals with high extraversion and low openness looked at the forehead. In evaluating neuroticism, individuals with high neuroticism tended not to look at the forehead, eyebrows, and eyes for an extended period. Furthermore, individuals with high conscientiousness did not look at the forehead and mouth. When assessing openness, individuals with high conscientiousness tended not to look at the mouth. Moreover, compared to the results of the free observation condition, the number of significant correlations notably increased.

**Table 4:** Significant correlations between eye movements and personality traits (Effective visual field condition).

Model	Impression	Area	Predictor	Mean	95%HDI
Bernoulli	Agreeableness	Forehead	Agree.	5.404	0.156 to 11.195
			Extra.	4.535	0.597 to 8.773
			Open.	− 6.420	− 13.138 to − 0.751
		Eyebrow	Agree.	15.161	3.904 to 27.740
			Neuro.	− 5.113	− 11.193 to − 0.316
		Eye	Agree.	15.197	3.915 to 27.818
			Neuro.	− 5.106	− 11.195 to − 0.002
	Conscientiousness	Forehead	Extra.	5.053	0.595 to 9.401
			Open.	− 6.474	− 13.772 to − 0.354
	Extraversion	Forehead	Agree.	2.013	0.222 to 3.830
			Consc.	− 2.910	− 5.796 to − 0.474
			Extra.	1.983	0.119 to 4.086
			Open.	− 1.335	− 2.569 to − 0.151
	Neuroticism	Forehead	Agree.	2.210	0.768 to 4.006
			Consc.	− 2.653	− 5.112 to − 0.433
			Extra.	1.906	0.461 to 3.611
		Eyebrow	Agree.	15.140	3.679 to 26.262
			Neuro.	− 5.042	− 10.816 to − 0.271
		Eye	Agree.	15.017	3.974 to 26.001
			Neuro.	− 4.995	− 10.601 to − 0.202
		Mouth	Consc.	− 0.863	− 1.619 to − 0.190
	Openness	Mouth	Consc.	− 0.661	− 1.267 to − 0.008
Beta	Agreeableness	Glabella	Neuro.	0.167	0.031 to 0.315
		Mouth	Open.	− 0.297	− 0.507 to − 0.085
	Conscientiousness	Glabella	Neuro.	0.217	0.060 to 0.371
	Openness	Glabella	Neuro.	0.177	0.040 to 0.309

#### Verify changes in eye movements through simulation

**Figure 6:**
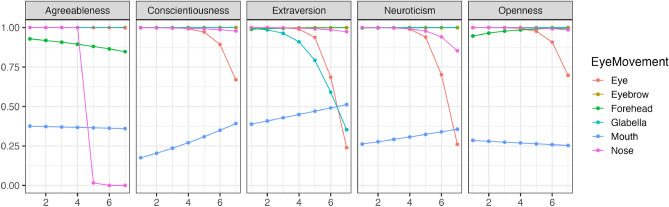
The impact of conscientiousness variations on eye movement during free observation condition (Bernoulli distribution). The x-axis represents the conscientiousness scores, and the y-axis shows the probability of focusing on each part as estimated by the model. The closer to 1, the higher the probability of focus.

**Figure 7:**
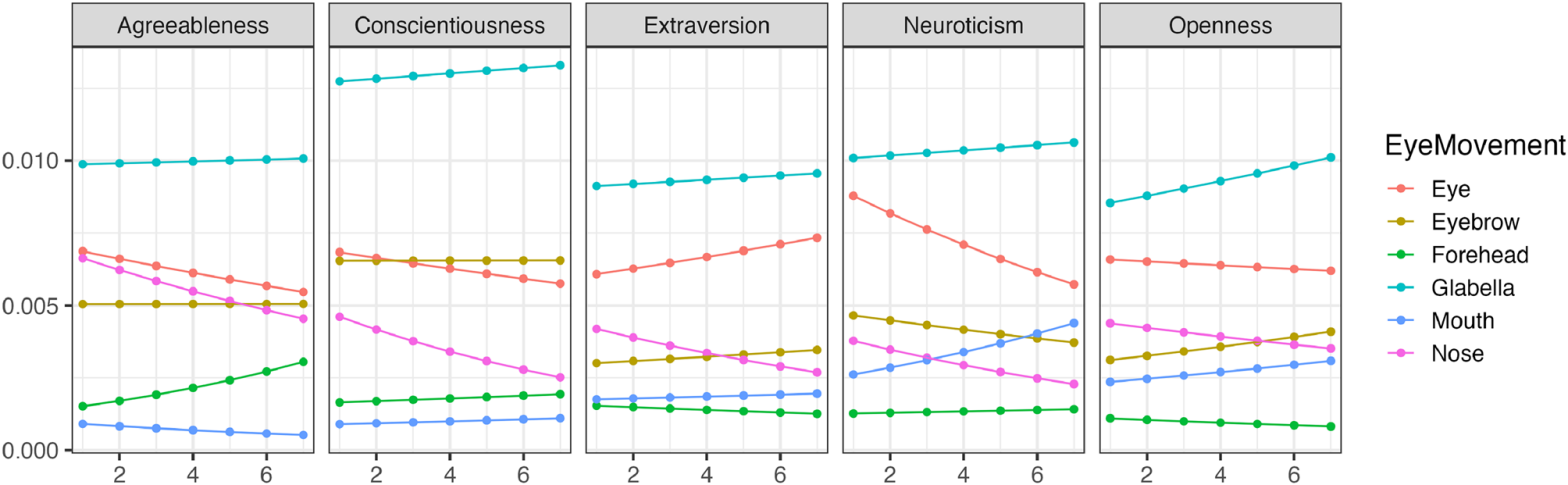
The impact of Conscientiousness variations on eye movement during free observation condition (Beta distribution). The x-axis represents conscientiousness scores, and the y-axis indicates the frequency of focus on each part as estimated by the model (following a beta distribution). The higher the value of Y, the higher the frequency of focus.

Using the parameters estimated by the model, we simulated the changes in eye movements for different impression ratings. Figure 6 illustrates the trend of changes in whether each area was observed (Bernoulli distribution) with variations in conscientiousness during free observation. Figure 7 shows the trend of changes in how much time was spent observing each area (Beta distribution) with variations in conscientiousness. Conscientiousness was rated on a scale of 1–7, whereas other personality traits were fixed at 3. The results indicated that except for agreeableness ratings, an increase in conscientiousness corresponded to a tendency not to look at the eyes during free observation. Moreover, in evaluating agreeableness, higher conscientiousness was associated with a tendency not to look at the nose. Though there was a partial increasing trend, participants generally did not focus on the mouth. However, except for neuroticism ratings, an increase in conscientiousness corresponded to a tendency to spend less time looking at the eyes.

**Figure 8:**
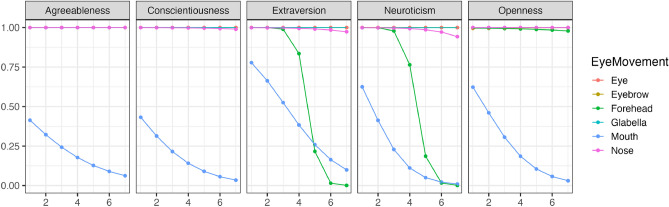
The impact of Conscientiousness variations on eye movement during discrimination visual field condition (Bernoulli distribution). The x-axis represents the conscientiousness scores, and the y-axis shows the probability of focusing on each part as estimated by the model. The closer to 1, the higher the probability of focus.

**Figure 9:**
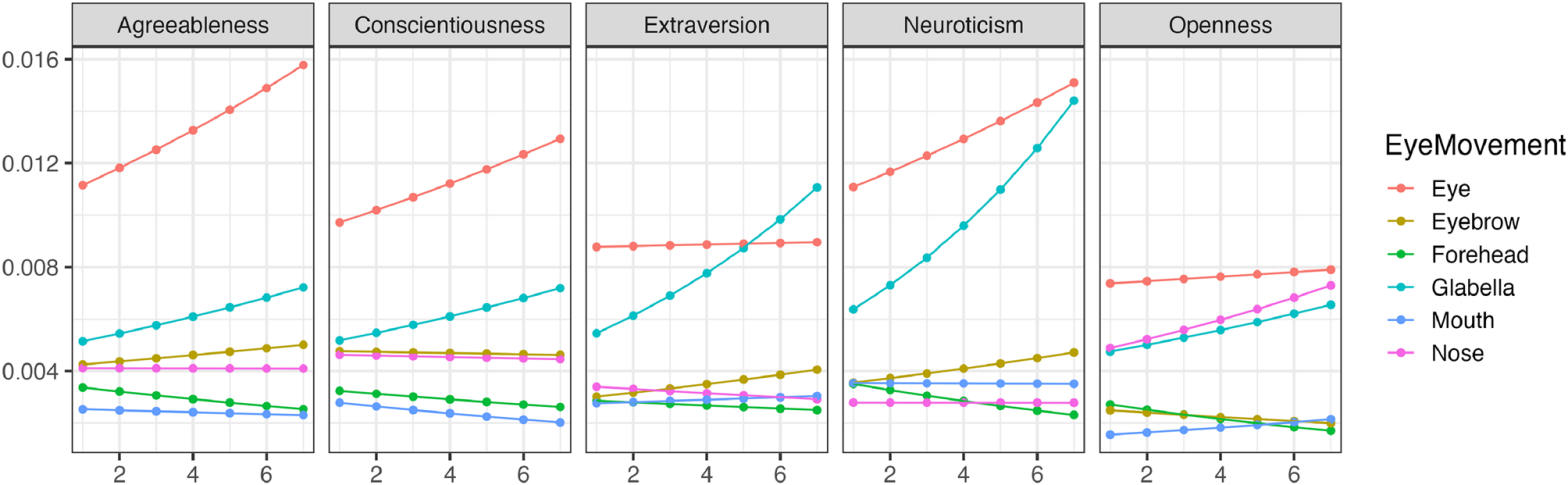
The impact of Conscientiousness variations on eye movement during discrimination visual field condition (Beta distribution). The x-axis represents conscientiousness scores, and the y-axis indicates the frequency of focus on each part as estimated by the model (following a beta distribution). The higher the value of Y, the higher the frequency of focus.

Figure 8 illustrates the trend of changes in whether each area was observed (Bernoulli distribution) with variations in conscientiousness when the visual field was restricted. Figure 9 shows the trend of changes in how much time was spent observing each area (Beta distribution) with variations in conscientiousness when the visual field was restricted. The results indicated that regardless of conscientiousness variations, participants consistently looked at the eyes, nose, eyebrows, and forehead with high probability while avoiding looking at the mouth with high probability in all impression ratings when the visual field was restricted. Nevertheless, in evaluating agreeableness, conscientiousness was associated with a significant increase in the duration of looking at the eyes and forehead as conscientiousness increased.

**Figure 10:**
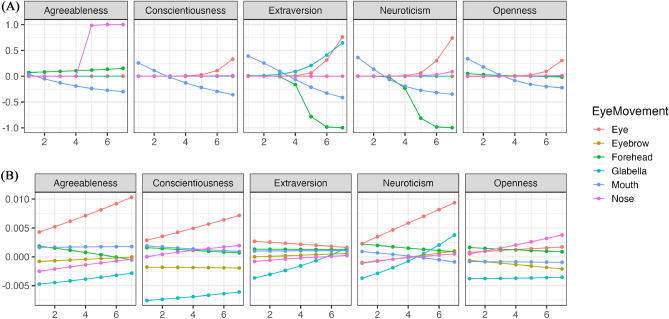
The relationship between the differences in estimated results between free observation and restricted visual field conditions and changes in conscientiousness. The x-axis represents the conscientiousness scores, the y-axis of A shows the difference in the probability of focusing on each part, and the y-axis of B shows the difference in the frequency of focusing on each part. If greater than 0, the restricted visual field condition is dominant; if less than 0, the free observation condition is dominant; the closer to 0, the less change there is.

To make the comparison between changes observed during free observation and restricted visual field conditions more understandable, the results from the simulations are displayed after performing subtraction (results of restricted condition–results of free condition). Panel (A) of Fig. 10 illustrates the difference between Figs. 6 and 8, while Panel (B) shows the difference between Figs. 7 and 9. A value greater than 0 indicates that the restricted visual field condition had a greater impact; a value less than 0 suggests that the free observation condition was more influential; the closer the value is to 0, the smaller the observed change. For more detailed results, please visit the provided https://osf.io/8zfc5/. According to the results in Panel (A) of Fig. 10, when assessing neuroticism and extraversion, individuals with higher levels of conscientiousness significantly increase their likelihood of focusing on the eyes in the restricted visual field condition, while their likelihood of focusing on the forehead decreases markedly. Conversely, when assessing agreeableness, these individuals are significantly more likely to focus on the nose in the restricted visual field condition. As shown in Panel (B) of Fig. 10, in assessments of agreeableness, conscientiousness, and neuroticism, individuals with higher levels of conscientiousness tend to spend significantly more time focusing on the eyes in the restricted visual field condition.

The estimated results of the impact of variations in other personality traits on eye movements can be reviewed in the appendix data (https://osf.io/8zfc5/). The estimated results of how much time was spent observing each area (Beta distribution) showed different patterns depending on the personality trait. For instance, unlike the trend of conscientiousness variations when the visual field was restricted, agreeableness, extraversion, and openness showed a tendency not to look at the eyes as the respective traits increased. However, the estimated results of whether each area was observed (Bernoulli distribution) also exhibited distinct patterns. For example, in contrast to the trend of conscientiousness variations when the visual field was restricted, agreeableness and extraversion showed a tendency to have a higher probability of looking at the mouth as they increased, respectively.

### Discussion

This study aims to extract conscious eye movements through visual field restriction and investigate their relationship with personality traits. Therefore, referring to previous studies, a behavioral experiment was conducted (Experiment 1) in which the visible range was set within the discriminative field of view. As a result, different eye movements were observed under free observation and visual field restriction conditions. Furthermore, different relationships between personality traits and eye movements were observed under each condition. In other words, under free observation conditions, a mixture of conscious and unconscious eye movements was suggested. It is unlikely that eye movements under these conditions perfectly represent true human observation behavior. To investigate consciously expressive eye movements that represent true intentions, it would be more straightforward and effective to limit the observation field to some extent. However, Experiment 1 showed limited significant correlations between personality traits and eye movements, and simulations generally indicated that participants were looking at each facial area regardless of the impression rating. This could be attributed to the possibility that the visible range was too narrow. To verify more accurate conscious eye movements, the size of the restricted range was adjusted, and Experiment 2 was conducted.

Experiment 2 set the boundary line slightly beyond the discriminative field of view, transitioning from the discriminative field of view to the effective field of view. The reason for this decision is the concern that if the visible range is too wide, the effect of range restriction may diminish, and conscious eye movements might not be extractable. As a result, under free observation conditions, similar results to Experiment 1 were obtained, but under restricted range conditions, a notable increase in the number of significant correlations between personality traits and eye movements was observed, showing a similar trend in simulations. In other words, the appropriate range for extracting conscious eye movements is the effective field of view, not the discriminative field of view. Notably, the effective field of view used in this experiment covers approximately two-thirds of the facial area in the stimulus images. Expanding the range further may weaken the effect of field restriction, potentially hindering the extraction of conscious eye movements.

Taking the change in conscientiousness as an example, interesting differences in eye movements between different scenarios were observed. Under free observation conditions, as conscientiousness increased, the probability of not looking at the eyes increased, while the probability of not looking at the mouth remained almost unchanged (Fig. 6). Under restricted range conditions, as conscientiousness increased, the probability of not looking at the mouth increased, while the probability of looking at the eyes remained almost unchanged (Fig. 8). These results suggest that during free observation, higher conscientiousness leads to a tendency for eye movements to concentrate within the face, observing the entire face. This tendency aligns with culturally specific observation behaviors demonstrated in previous studies, such as the focus on the nose observed in East Asian participants^14^. On the other hand, under restricted range conditions, focusing on specific areas consciously, there was a higher and sustained probability of looking at the eyes and nose compared to the mouth. In other words, observational behavior in a consistently restricted range scene may better represent a person’s conscious eye movements. Additionally, the results revealed discrepancies in the stay frequency of eye movements between free observation and visual field restriction conditions. The restricted range condition showed a clear increase in the frequency of focusing on the eyes (Fig. 10).

When further comparing the results of Experiment 2, an interesting observation emerged. During free observation, significant attention was allocated to various facial features, such as the eyes, nose, and mouth. However, in scenarios where the visual field was restricted to the effective field of view, attention was significantly focused only on the upper parts of the face (eyes, forehead, eyebrows, and glabella). These findings resonate with two insights from previous research. The first insight is the tendency of East Asians to concentrate their gaze on the central part of the face^14,33,49^. The second insight highlights the instinctual awareness humans have towards the eyes or gaze direction of others^50,51^. Thus, the reason East Asians may not directly observe the eyes (or maintain gaze) when looking at faces is not due to a lack of awareness of the eyes but rather to a combination of unconscious eye movements influenced by culture or education. By identifying and accounting for such unconscious eye movements, we can reveal the true relationship between personality traits and eye movements.

## Conclusion

This study focuses on the relationship between conscious eye movements induced by visual field restriction and individual personality traits. We hypothesized that under the constraints of visual field restriction, individuals would allocate visual attention and move their eyes differently, based on their personality traits. To elucidate this relationship, Experiments 1 and 2 were conducted. The results revealed that restricting the observation area to a moderate extent (effective visual field) made it possible to capture individuals’ conscious eye movements. Interestingly, even though all participants were East Asians, it was found that conscious eye movements primarily focused on the upper part of the face (eyes, eyebrows, etc.). Furthermore, significant correlations between personality traits and conscious eye movements presented different results from those observed under free observation conditions. These findings not only support previous insights but also propose a novel research design for exploring the relationship between personality traits and eye movements. Nonetheless, the study leaves some questions unanswered. For example, differences in the results of the free observation conditions between Experiments 1 and 2 were noted. This discrepancy might be attributed to variations in the personality traits of participants across both experiments. Future research will need to accurately assess individual differences using a more balanced sample. In conclusion, visual field restriction successfully simplified complex eye movements, indicating that these movements are influenced by individual personality traits. Through detailed data collection and analysis, future research aims to deepen our understanding of how specific personality traits influence visual behavior, with the anticipation of contributing to future applications and psychological assessments.

## Data Availability

Our datasets, stan model and R code generated during this study are available on OSF at https://osf.io/8zfc5/.
